# Broad impacts of fine-scale dynamics on seascape structure from zooplankton to seabirds

**DOI:** 10.1038/ncomms6239

**Published:** 2014-10-15

**Authors:** Arnaud Bertrand, Daniel Grados, François Colas, Sophie Bertrand, Xavier Capet, Alexis Chaigneau, Gary Vargas, Alexandre Mousseigne, Ronan Fablet

**Affiliations:** 1 Institut de Recherche pour le Développement (IRD), UMR212 EME IFREMER/IRD/UM2, Avenue Jean Monnet, CS 30171, Sète Cedex 34203, France; 2 Instituto del Mar del Perú, Esquina Gamarra y Gral, Valle s/n, Callao 05, Peru; 3 IRD-CNRS-Sorbonne Universités (UPMC Univ. Paris 6)-MNHN, LOCEAN/IPSL Laboratory, 4 Place Jussieu, Paris 75252, France; 4 IRD, UMR LEGOS, CNES/CNRS/IRD/UPS, 14 Avenue Edouard Belin, Toulouse 31400, France; 5 TELECOM Bretagne, UMR CNRS-3192-Lab-STICC, Brest 29238, France

## Abstract

In marine ecosystems, like most natural systems, patchiness is the rule. A characteristic of pelagic ecosystems is that their ‘substrate’ consists of constantly moving water masses, where ocean surface turbulence creates ephemeral oases. Identifying where and when hotspots occur and how predators manage those vagaries in their preyscape is challenging because wide-ranging observations are lacking. Here we use a unique data set, gathering high-resolution and wide-range acoustic and GPS-tracking data. We show that the upper ocean dynamics at scales less than 10 km play the foremost role in shaping the seascape from zooplankton to seabirds. Short internal waves (100 m–1 km) play a major role, while submesoscale (~1–20 km) and mesoscale (~20–100 km) turbulence have a comparatively modest effect. Predicted changes in surface stratification due to global change are expected to have an impact on the number and intensity of physical structures and thus biological interactions from plankton to top predators.

## Abstract (web summary)

Ocean circulations can strongly influence ecological dynamics in marine ecosystem over multiple spatial scales. Here, Bertrand *et al.* find that the majority of these interactions occur within small-scale hotspots that concentrate interactions across many trophic levels from zooplankton to seabirds.

## Graphical abstract

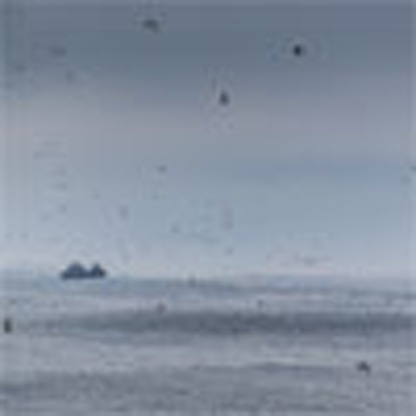

Living organisms follow non-random yet non-uniform distributions and tend to aggregate in patches^1^,^2^. Both physical forcing and organism behaviour are implicit in the initiation and maintenance of this patchiness, with the latter increasing in importance with each step up the trophic chain^3^. Physical forcing initiates the structuring of the water masses and the distribution of planktonic organisms. Since predators are required to locate their prey, their foraging behaviour tends to reflect the patchy distribution of their prey^1^,^3^.

Physical forcing results from a myriad of turbulent processes that span a wide range of scales and influence organism distribution and behaviour in a variety of ways. At the fine scale (~1–10 m vertically), the importance of thin layers has recently been emphasized owing to their ubiquitous nature and their potential to induce ecological hotspots and increase trophic transfer rates from phytoplankton to higher trophic levels^4^. At broader (horizontal) scales, strong evidence suggests that internal wave (IW; ~100 m to 10–15 km), submesoscale (~1–20 km; for example, fronts and filaments) and mesoscale (~20–100 km; for example, eddies) activity modulates the concentration and distribution of marine organisms, thereby influencing ecosystem dynamics^5^,^6^,^7^,^8^. Observations on fine scale (~100 m–10 km, horizontally) dynamics do exist (for example, refs 9, 10) and the associated studies stress the impact on primary productivity enhancement, plankton transport and aggregation of predators of several trophic levels (for example, refs 11, 12, 13). Despite their relevance, they focused on specific areas and could not facilitate the development of comprehensive ecosystem-level models from physics to top predators. Along with computational complexity, these elements explain why the incorporation of these processes in models of marine ecosystem dynamics is still in its infancy, particularly at scales below the mesoscale.

Underwater acoustics have an unrealized potential for multicomponent observations that can overcome previous limitations. Only underwater acoustics make feasible the simultaneous collection of qualitative and quantitative data on the distribution and behaviour of various communities of an ecosystem, from plankton to large predators as well as abiotic parameters at a variety of spatiotemporal scales^14^. Here we use a unique data set to explore the multiscale distribution of turbulent structures (for example, IW or eddies) and of their aggregative strength over three trophic levels and across scales from 100 m to 30 km. Data were collected in the Northern Humboldt Current System (NHCS) off Peru, the marine ecosystem the most impacted by climate variability and the most productive in terms of fisheries^15^. It encompasses an intense and shallow oxygen minimum zone (OMZ), which structures the ecosystem vertically^16^. Simultaneous high-resolution information (40 m grain scale over 18,000 km survey track) on the lower oxycline location^16^, reflecting upper ocean dynamics (see Methods), and on zooplankton and pelagic fish biomass distribution (Fig. 1a) was extracted from acoustic data^17^. Information on the space occupancy of top predators was obtained from global positioning system (GPS)-tracking data (see Methods) on Peruvian boobies *Sula variegata* and Guanay cormorants *Phalacrocorax bougainvillii*, the most abundant piscivorous seabird species in the NHCS^18^.

## Results and Discussion

### Multiscale patterns of ocean turbulence

**Figure 1:**
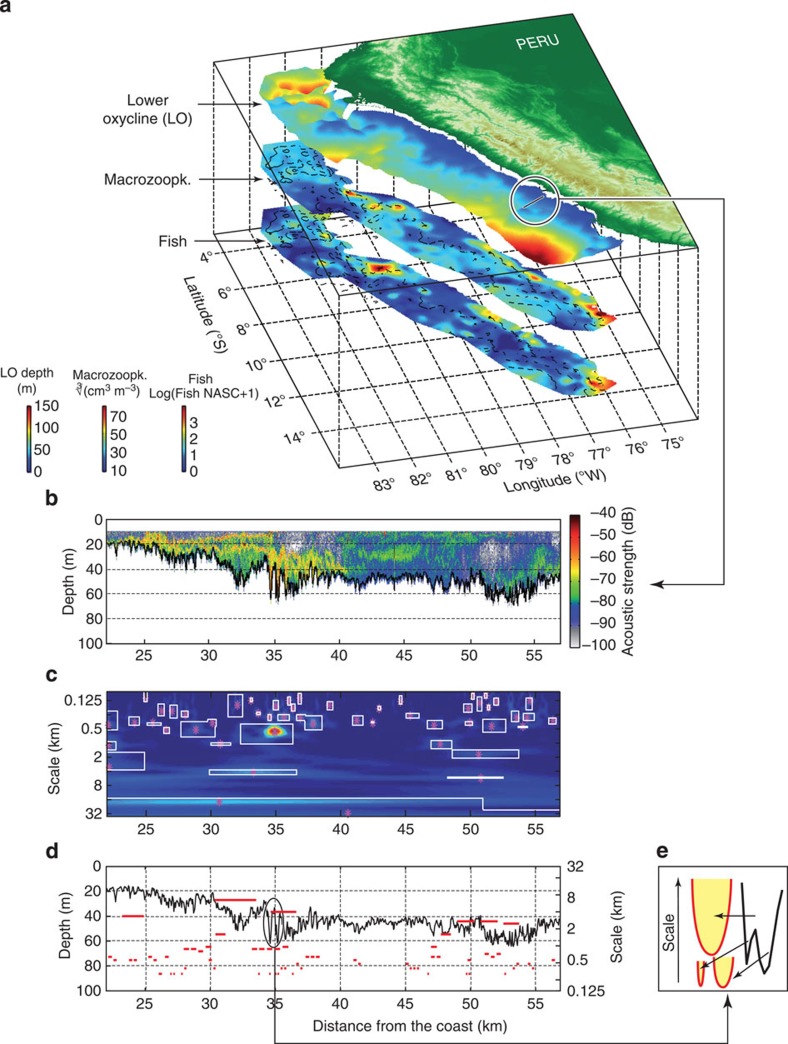
Data description. (**a**) Upper volume: acoustically estimated lower oxycline (in m). Intermediate surface: zooplankton biovolume above the lower oxycline. Lower surface: fish biomass above the lower oxycline. (**b**) Acoustic echogram along a given transect (see **a**) with the lower oxycline (black solid line). (**c**) Power wavelet spectrum of the lower oxycline depth from **b**; white rectangles correspond to significant space-scale local maxima. (**d**) Lower oxycline depth (black solid line) and significant structures (red lines) extracted from **c** and mapped according to their characteristic scale along the right *y* axis. (**e**) Expansion from **d** showing a section of the lower oxycline (black solid line) and significant extracted space-scale structures (red lines) and their corresponding downward DS (yellow areas). The plots depict daytime data recorded during austral spring of 2010.

Turbulence is a dynamic process that can be tracked through the deformations it generates in observable water mass properties, such as the vertical deformations of the oxycline or the pycnocline. Here we analysed the depth of the lower oxycline as observed by acoustics (Fig. 1b–e) and performed a wavelet analysis^19^ to identify and characterize the multiscale patterns of ocean turbulence from ~100 m to ~100 km. This relies on the extraction of dominant scale-space structures and on the estimation of the associated downward deformation surface (DS, in m^2^ see Fig. 1e). Our diagnosis focuses on the downward displacements (that is, deformation) of the lower oxycline induced by dynamical processes as they are by far the most commonly observed.

**Figure 2:**
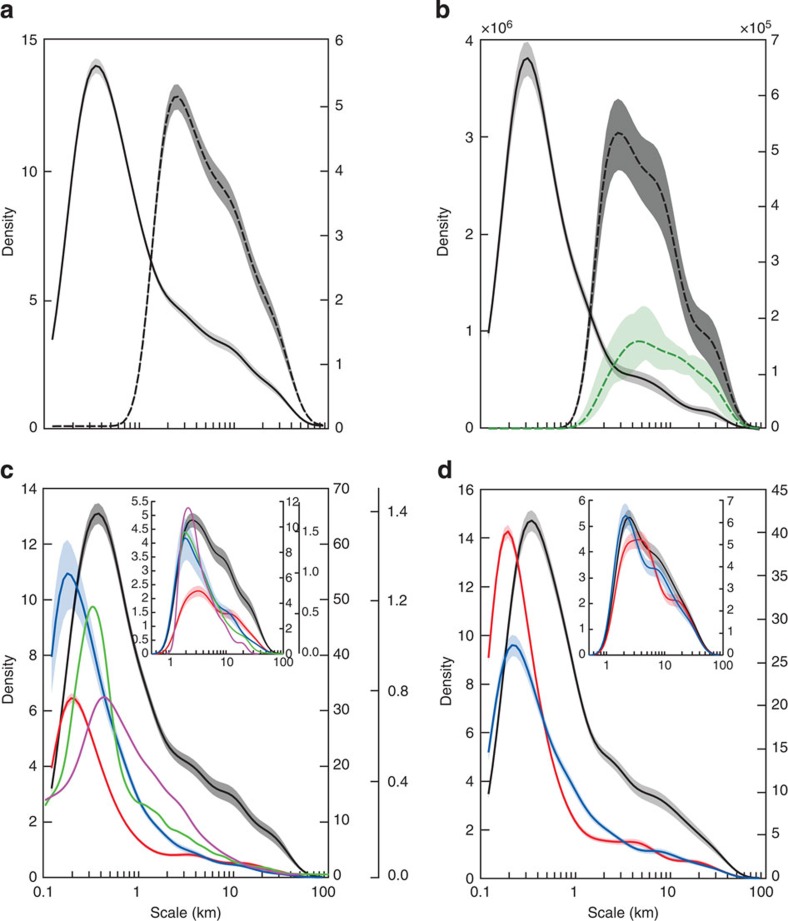
Space-scale patterns. (**a**) Density function (normalized) of the cumulative downward DS (in m^2 ^m^−1^) of space-scale structures extracted from the lower oxycline with a grain scale of 500 m (black dashed line) and 40 m (black solid line). (**b**) Equivalent to **a** with a grain scale of 40 m (black solid line) for the conditions corresponding to the ROMS model configuration (November month) along with the density function of the DS extracted from the modelled pycnocline depth (green dashed line; grain scale: 500 m). In **a**,**b**, the left and right *y* axes correspond to grain scale of 40 and 500 m, respectively. (**c**) Daytime density function (normalized) of the cumulative DS (black), zooplankton biovolume (red solid line) and fish biomass (blue) of the space-scale structures (grain scale: 40 m) and density function of the ARS sizes of Peruvian booby (green) and guanay cormorant (pink). Inset plot focuses on structures larger than 1.5 km. Left *y* axis corresponds to DS, the first right *y* axis corresponds to zooplankton and fish, while the second right *y* axis corresponds to seabirds. (**d**) Equivalent to **c** but for nighttime data. Shaded areas represent the confidence intervals.

When using the high-resolution acoustic data (40 m; ~15,000 identified structures), a dominant peak emerges in the DS density spectrum at *O*~300 m (Fig. 2a, Supplementary Table 1). The shape of the identified structures strongly suggests that this DS peak is due to IW activity. Packets of solitary waves with amplitudes of 20–50 m and shapes consistent with those of solitons were extremely common in our echograms (Fig. 1b), similar to many coastal regions^9^,^20^. The archetypal generation process for IW activity^21^,^22^ involves the interaction between a barotropic tidal flow (of moderate intensity off Peru) and an abrupt topography. As a consequence, the shelf break and outer shelf region (where the overwhelming fraction of the structures corresponding to the *O*~300 m peak is found; Supplementary Fig. 1) tend to be regions of intense IW activity.

We observed a conspicuous shoulder pattern on the DS spectrum (that is, a rupture in the slope of the decay of the DS spectrum) for scales above 2–3 km (Fig. 2a). Using a larger grain scale (500 m), the shoulder pattern becomes a well-defined peak coinciding with the submesoscale range (~3 to ~10 km). To support its interpretation, a Regional Ocean Modelling System^23^ (ROMS) configuration was run with a 500-m horizontal grid resolution (see Methods and Supplementary Fig. 2). In this simulation, forcing sources of IW and high-frequency processes (tides and high-frequency content of atmospheric forcing) were absent. At such horizontal scales, the model is known to produce realistic levels of submesoscale turbulence^24^. We performed wavelet analysis on both the observed lower oxycline and the pycnocline depth modelled by ROMS at the same grain scale (500 m) in a comparable domain (Fig. 2b and Supplementary Fig. 2). DS from acoustic data and ROMS outputs exhibit similar patterns in their density functions (Fig. 2b). The peak of model simulation data at *O*~3 km is consistent with the presence of abundant submesoscale features (for example, fronts, small coherent eddies; Supplementary Figs 2 and 3) generated by flow instabilities^25^. Model estimates of the mixed-layer internal Rossby radius, a typical horizontal scale for mixed-layer instabilities and turbulence^26^, are in the range 3–7 km. Yet, for scales <10 km, DS values from acoustic observations are two to three times higher than those from the model. This strongly suggests that IW and high-frequency processes, which are not accounted for in the model, remain dominant over the submesoscale range^27^. At larger scales ~15 km and above, subinertial turbulent processes are expected to be increasingly dominant over IW ones, and ROMS DS levels are similar to those computed for observation data. The first baroclinic Rossby radius of deformation in the nearshore NHCS typically results in mesoscale structures or coastally trapped waves at the scale of ca 30 km (ref. 28) and could explain the DS shoulder patterns we observed at the largest scales we resolved.

Overall, our findings indicate that oxycline deformations are primarily driven by small-scale processes (<1 km), related to short IW activity. Nonetheless, a significant portion of oxycline deformations remains associated with submesoscale activity (Fig. 2a). Submesoscale dynamical processes may only account for 30–50% of these small-scale deformations (Fig. 2b).

### Multiscale patterns of organisms space use

**Figure 3:**
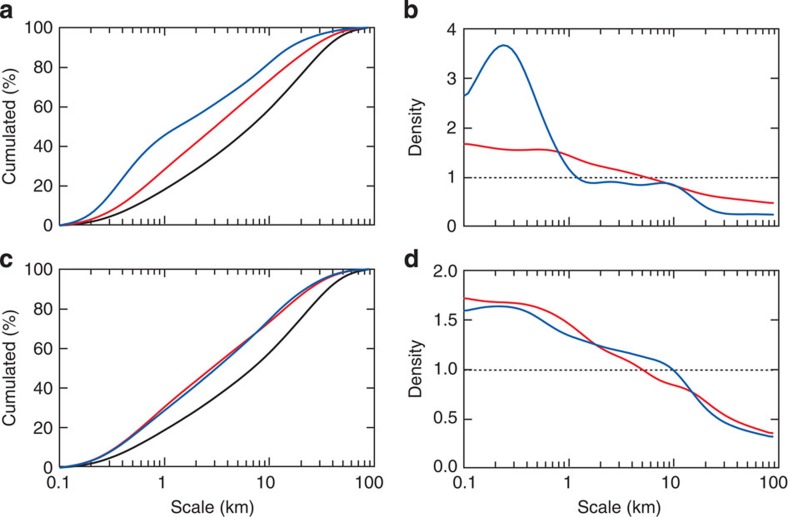
Cumulative biovolume/biomass within DS space-scale structures. (**a**) Standardized cumulative downward DS (black), and zooplankton (red) and fish (blue) biomass embedded in the corresponding space-scale structures during the day (**c** depicts nighttime data); grain scale: 40 m. (**b**) Ratio between the density function of the cumulative embedded zooplankton (red) and fish (blue) biomasses and the density function of cumulative DS (**d** for nighttime).

A wavelet-based analysis was similarly applied to the distribution of living organisms. Significant scale-specific patches were extracted from both zooplankton biovolume and fish biomass along transects. In addition, area-restricted searches^29^ (ARS) were extracted from seabird foraging tracks (Fig. 3c,d) obtained using GPS^30^. ARS associated with favourable forage environments typically form nested patterns at scales ranging from a few metres to kilometres^30^ and indicate a behavioural response to meso- or submesoscale^6^,^31^ structures.

The scale-space structures found for zooplankton, fish and seabirds matched those of ocean dynamics (Fig. 2c,d; Supplementary Table 2). The peak (estimated from the zero crossings of the second derivative of the density function of the process of interest; Supplementary Tables 1 and 2) at *O*~300 m across all three trophic groups largely reflected the characteristic scale range of IW activity. According to ref. 32, this scale range corresponds to a level of patchiness (for example, fish shoals), for which both environment (biotic and abiotic) and self-organization (social behaviour) processes are critical. Interestingly, here we show that at the smallest scales, the size of seabird structures lines up with those of the physics, while the fish and zooplankton patches peak at slightly smaller scales (Fig. 2c,d; Supplementary Table 2). This suggests that, while seabirds use IWs as foraging cues^11^ (producing ARS scales fitted closely to those of IW), zooplankton and fish patches are embedded within IW^5^ under the combined influence of flow patterns in the vicinity of IWs and the increased role for behaviour and active movements at smaller scales^32^.

Two peaks were observed at the submesoscale (one at *O*~3 km and a second one at *O*~9 km) and one at the mesoscale (*O*~20–30 km; insets in Fig. 2c,d; Supplementary Table 2), which again mirrored observations of ocean turbulence. Fish and zooplankton peaks were stronger at night when vertical diel migrations cause organisms to concentrate above the lower oxycline^16^,^17^ as observed during a small-scale *in situ* experiment in the NHCS^32^.

### Seascape and behaviour shape organisms’ distribution

To assess the extent to which fine-scale turbulence concentrates organisms, we investigated the scale-related aggregative power of physical processes. First, we compared zooplankton and fish densities and biomasses within and outside of (the nearest space not forming part of any structure) identified structures. The results of this comparison provide evidence of significant ocean-dynamic-driven aggregation (Table 1). The mean zooplankton and fish densities were, respectively, 8.8% and 92% higher within structures. These structures are characterized by downward vertical deformations, which provide oases with a larger volume of habitat and a greater organism density. This led to concentrating biomass with the mean zooplankton and fish biomasses, respectively, ~150% and ~950% higher within structures than in the neighbouring zone with no significant physical structures (Table 1). The stronger aggregation for fish than zooplankton suggests that behaviour, that is, schooling and the search for prey, can magnify physically induced spatial structuring. Second, by comparing organism aggregations among structures we found that 30–45% of zooplankton biovolume and fish biomass were embedded within structures smaller than *O* (1 km), which accounted for less than 20% of the total DS (Fig. 3). As such, IW processes have a stronger effect on organisms’ distribution relative to the magnitude of the vertical deformation. This effect, although still existent, is not as strong for submesoscale structures (<10 km).

**Figure 4:**
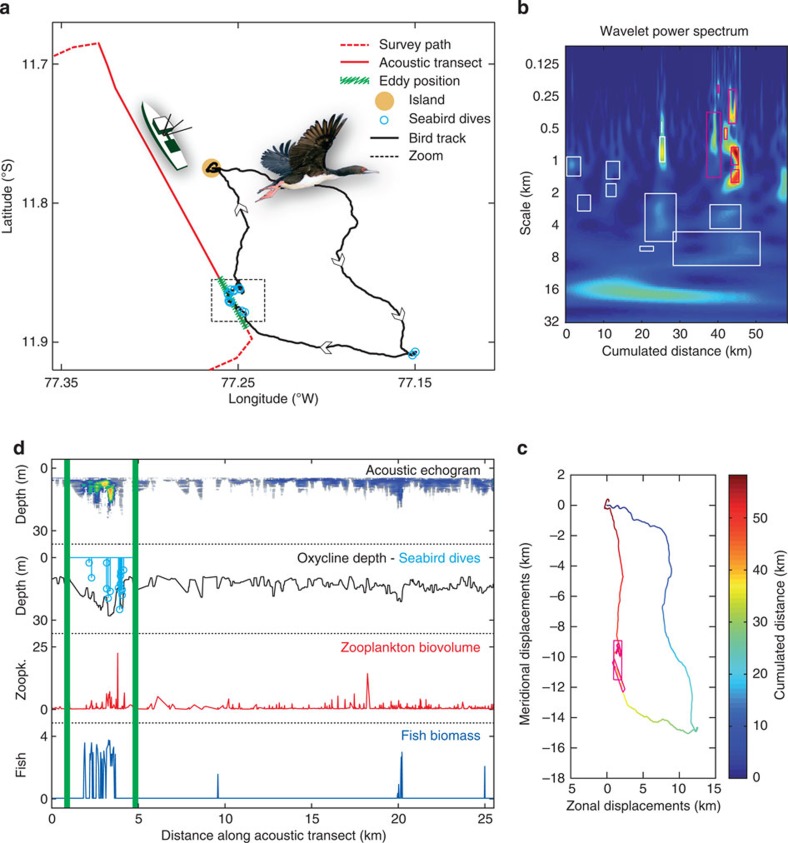
Example of the bottom-up structuring transfer. (**a**) Research vessel survey path (red) on 25 November 25 2011 and simultaneous GPS track of a guanay cormorant (black); the blue dots refer to seabird dives, whereas the green line delimits the structure described in **d**. (**b**) Wavelet spectrum of the seabird trajectory with significant ARS (white and red rectangles). (**c**) Seabird trajectory with ARS (red rectangles) in the area illustrated in **d**. (**d**) Acoustic transect (red solid line in **a**) with the corresponding acoustic echogram, lower oxycline and seabird dive profiles, zooplankton biovolume (in 
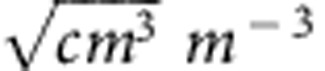
) and fish biomass (in log(NASC+1)). A submesoscale structure of 2.6 km long was observed, which we related to a coherent eddy. Two smaller structures (1,000 and 360 m long) were embedded within it, presumably nonlinear IW. Seabird-diving patterns coincide remarkably well with the shape of the submesoscale feature as well as with the depth of the lower oxycline. Horizontally, the diameter of the largest ARS matching the spatial position of the physical structure was 1.7 km. Smaller ARS were embedded within this larger ARS with sizes varying between 320 m and 1.4 km that mirror lower oxycline displacements at similar scales.

The observed bottom-up transfer through the food web is clearly illustrated in Fig. 4. A GPS-tracked guanay cormorant went across the research vessel survey path (see Supplementary Note 1). Acoustics revealed the presence of a submesoscale structure of 2.6 km long akin to a coherent eddy that aggregated zooplankton and fish. The seabird actively foraged within this structure and its diving patterns remarkably coincided with the shape of the submesoscale features as well as with the depth of the lower oxycline.

### Synthesis

In summary, we have shown that physical and biological fields, across trophic levels, share multiscale aggregative patterns at scales ranging over two orders of magnitude (300 m–30 km). While forage fish and predators were known to concentrate at hotspots, we demonstrate that important levels of aggregation occur at finer scales. Primary ecosystem interactions were believed to occur at meso- or submesoscale^6^,^7^,^8^. However, we show that the majority of interactions most likely occur within small, short-lived physical structures that create small-scale (*O*<1–4 km) ephemeral hotspots, which concentrate organisms ranging from zooplankton to seabirds. The acoustic methodology used here can be applied to other marine ecosystems even in the absence of an OMZ. IW, submeso- and mesoscale features are commonly observed in echograms from several oceanic regions^9^,^10^. These can be easily monitored to further understand and evaluate this multiscale, bottom-up structuring of marine ecosystems. The intensification of upper ocean stratification resulting from global change^33^ makes such high-resolution analyses even more critical, given the expected impacts on both the number and intensity of physical structures and, consequently, on distribution patterns of marine life and resultant trophic interactions.

## Methods

### Acoustic data

Acoustic data were collected on board the 41-m-long research vessel (R/V) ‘Jose Olaya’ from the Instituto del Mar del Perú (IMARPE) during four routine scientific surveys performed in February to April 2006, November to December 2008, February to April 2010 and November to December 2010. Hull-mounted Simrad split-beam bi-frequency (38 and 120 kHz) scientific echo-sounders EK500 and EK60 (Kongsberg Simrad AS) were used to acquire the data with an average ping rate of 1 s. Survey tracks consisted of parallel cross-shore transects with a typical vessel speed of 4.5±0.4 m s^−1^. Echosounder calibration was performed according to ref. 34. The water column was sampled down to depths of 250 and 500 m for the 120 and 38 kHz frequency channels, respectively. Owing to the presence of noise in echograms at 120 kHz, only the first 150 m were considered. Only day and night periods were considered; data from twilights were removed before analyses since it is not possible to determine oxycline depth when mesopelagic organisms migrate through the upper limit of the OMZ^16^.

### Acoustic scatter discrimination

Acoustic data used in this work were processed using a bi-frequency method developed by refs 16, 17. Data processing was performed using Echoview (SonarData Pty. Ltd., Hobart, Tasmania, Australia) and an open source software Echopen ( http://www.france-nord.ird.fr/les-ressources/outils-informatiques) developed in Matlab (MathWorks, Natick, MA, USA). The method is based on the acoustic properties of organisms and their theoretical frequency dependence^35^,^36^,^37^,^38^. The detailed methodology is described in ref. 17 and is based on the combination of the sum of the mean volume backscattering strength between the two frequencies (+MVBS_120+38_) and the difference between these frequencies (ΔMVBS_120−38_). Here three groups of organisms were defined when discriminating between scatters:

- The ‘total fluid-like’ group, referred to as zooplankton in this study. It is composed of zooplanktonic organisms with weakly scattering tissue and acoustic properties similar to the medium that are usually named ‘fluid-like’ zooplankton^39^. With the setting used in this study, the ‘fluid-like’ or ‘zooplankton’ group corresponds mainly to organisms larger than ~2 mm, in particular euphausiids and large copepods, but also encompasses other large crustacean macrozooplankton (for example, the squat lobster *Pleuroncodes monodon*, squilla larvae, galatheid and other decapod larvae) and gelatinous without gas bladders (for example, salps and siphonophores);
- The ‘fish’ group; in the NHCS, all exploited fishes (anchovy *Engraulis ringens*, jack mackerel *Trachurus murphyi* and mackerel *Scomber japonicus*) and most mesopelagic fishes are swimbladder-bearing. Therefore, any reference to ‘fish’ in this study pertains to swimbladder-bearing fish. This group is highly dominated inshore by anchovy and offshore by mesopelagic fish in night data (see below);
- The ‘others’ group (‘blue-noise’ in ref. 17) composed of scatters not encompassed in the two groups described above. This group includes all targets other than fluid-like zooplankton and fish (mainly fish larvae, gelatinous and gas-bearing siphonophores).

Note that scattering from physical microstructures (for example, generated by IW turbulence) can have similar scattering level than zooplankton or fish over a range of frequencies^40^. Scatters originated by microstructures may have thus been misclassified towards fish or zooplankton. However, this problem was very unlikely to affect our results, since scattering is considered as mostly dominated by organisms^40^. Organisms scattering dominance are expected to be particularly strong in the NHCS, which is characterized by very high zooplankton and fish biomasses^15^,^17^.

### Oxycline estimation and relationship with the pycnocline

The Peruvian coast encompasses one of the world’s most intense and shallow OMZ that strongly structures the ecosystem^16^,^41^,^42^,^43^. The oxycline, which delimits the top of the OMZ, forms a sharp barrier for living organisms intolerant to hypoxia. Below, the upper part of the OMZ is generally almost free of organisms. The depth of the upper OMZ (lower oxycline depth) can be estimated from the depth of the vertical extension of the epipelagic community^16^. This method allows for a precise and robust determination of the upper limit of the OMZ with high spatiotemporal resolution. The robustness of the lower oxycline estimation is independent from the position from the coast (inshore versus offshore), the fish and zooplankton community structure and biomass, and the diel period (but not achievable during twilights when organisms migrate across the oxycline).

A subset of 26 density profiles for which the CTDO track was visible on the concomitant echogram^16^ was used to investigate the relationship between density and oxygen changes with depth. Supplementary Figure 4 represents the corresponding 26 profiles for


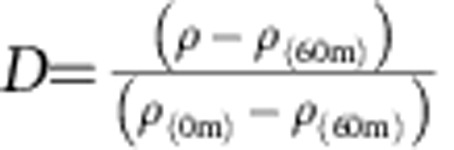
 as a function of z*=*z*−*z*(*D*=0.5). *D*=0.5 matches the region of largest vertical density gradient, that is, the pycnocline location. The definition of z* is such that adiabatic displacements, for example, induced by IWs are absorbed in the representation of *D* profiles in Supplementary Fig. 4. The position of the lower oxycline inferred from acoustics is also shown (black dots). Except in one case, they closely track the pycnocline with *D* differences between them of the order of 10–15 m or less (root mean square=7.4 m). Primary production or remineralization can certainly introduce some decoupling between density and oxygen spatial variability. Situations where such decoupling occurs on submesoscales (for example, owing to mixed-layer depth modulation by fronts) have been recently reported^44^. In the near-coastal NHCS we expect nonetheless that concomitant/concurrent displacements of the oxycline and pycnocline induced by energetic IW activity and submesoscale turbulence dominate on horizontal scales of metres to kilometres (Supplementary Fig. 4), which justifies our approach.

### Acoustic data used in analyses

The acoustic data used in this study were composed of three fields (for more details see refs 16, 17):

- the depth of the acoustically estimated lower oxycline depth. It is considered as a proxy of the structuring of the surface layer by dynamical processes^16^,^44^,^45^;
- the zooplankton biovolume (in mm^3 ^m^−2^) present in the oxygenated surface layer above the oxycline;
- the fish acoustic nautical-area scattering coefficient (NASC, an index of fish biomass in m^2 ^nm^−2^, see ref. 38) present in the oxygenated surface layer above the oxycline.

We only consider organisms present in the surface-oxygenated layer. Day and night organism data were evaluated separately to account for diel vertical migration pattern^17^. Since part of the zooplankton and mesopelagic fish perform a diel vertical migration, the daytime data comprise epipelagic organisms only, while night data correspond to both epipelagic and migrant organisms.

Acoustic data were collected at an average frequency of one ping per second, which corresponds to a ~4.5-m resolution along the vessel track. To remove high-frequency noise, we re-sampled the data according to 40-m-long elementary sampling distance units. Figure 1 shows the distribution of the acoustic fields issued from day data acquired in Austral Spring 2010 (see Supplementary Fig. 5 for corresponding night data).

### Seabird GPS tracks

Foraging seabird trajectories were collected between 2007 and 2012 on individuals breeding in two Peruvian coastal islands in austral spring (November to December): Guañape Sur Island (2007; 08**°**33′S; 78**°**58′W; ~12 km from the coast) and Pescadores Island (2008–2012; 11°46′S; 77°15′W; ~7 km from the coast). We fitted miniaturized GPS devices (Gipsy GPS, 25–30 g, Technosmart, Rome, Italy; and i-gotU GPS GT 600, 25–30 g, Mobile action Tecnology, New Taipei City, Taiwan) to 128 Peruvian boobies (*Sula variegata*) and 43 Guanay cormorants (*Phalacrocorax bougainvillii*). The GPS recorded locations at 1-s intervals and were attached with Tesa tape on the tail feathers for boobies and on the back feathers for cormorants for a number of days (usually 1 or 2). Some seabirds were also fitted with Time Depth Recorders (TDRs, G5, 3 g, CEFAS Technology, UK) attached to a metal leg band with Tesa tape. TDRs were programmed to record depth at 0.1-s intervals when submerged. Hydrostatic pressure data were corrected for surface drift. In Fig. 4a, dive was considered to occur only if hydrostatic pressure indicated a depth >2 m during more than 3 s.

### ROMS modelling

To help the interpretation of the observational results we used a realistic numerical simulation of the ROMS hydrodynamics ocean model^23^. The model was configured to represent the upper ocean submesoscale turbulence (horizontal mesh size of 0.5 km) in the central Peru region^46^. This submesoscale resolution model simulation was obtained using successive horizontal grid offline nesting refinements^47^ from the parent grid (horizontal mesh size of 7.5 km), encompassing the whole Southeastern Pacific, to child grids with 2 and 0.5 km resolutions. The downscaled model solution is for 1994–1995, a period considered to be close to normal conditions (for example, not subject to interannual El Niño/La Niña events). Details on the parent solution are described in ref. 48. Note that even though the model solution period does not correspond to the observational period, we believe that it is still useful because our purpose is to statistically characterize submesoscale processes in the NHCS and not to perform an exact comparison.

We used 10 fields of ROMS pycnocline depth outputs extracted from the 10-day simulation corresponding to spring conditions (November month; Supplementary Fig. 2). See Supplementary Fig. 3 for a snapshot of modelled sea surface temperature (in °C) exhibiting submesoscale coherent eddies and filaments off the coast of central Peru. In order to compare acoustic and ROMS data results, we used data with a similar spatial position. For that, we used the survey track trajectory to extract along transect data from the ROMS fields with an effective resolution of 500 m (Supplementary Fig. 2).

### Extraction of significant space-scale structures

In order to extract and characterize the significant variabilities of the physical processes across scales, we performed a wavelet-based analysis^20^ of physical data (namely, the lower oxycline depth observation data and the pycnocline depth signals from the numerical simulations). Given a signal *x*, we proceed according to a three-step procedure as follows:

Step 1: the wavelet spectrum of signal *x* is computed as the magnitude of the complex Morlet wavelet transform of *x* (ref. 19). Within this scale-space representation, the more energetic a given space-scale region is, the greater the variability around the considered time/space position at the considered scale range (Fig. 1b).

Step 2: the observed signal *x* was decomposed as the sum of a red noise process and of the signal of interest. To calibrate the parameters of the red noise, we used a LOESS (LOcally weighted Scatterplot Smoothing) smoothing function to detrend the signal and achieved a robust least trimmed square estimation of the s.d. and autocorrelation of the red noise^49^,^50^. We then defined the energy level in the wavelet spectrum above which a local space-scale is regarded as significant, as the 90th percentile of the wavelet spectrum of the red noise^19^.

Step 3: we detected the significant space-scale structures within signal *x* as the space-scale regions of the wavelet spectrum of *x*, which depict energy levels above the energy levels of the white noise model^30^.

The extracted structures may correspond to a variety of features such as IWs, coherent eddies and other. Each significant space-scale structure was characterized by a space window and scale range (See Fig. 1d). For each structure, we measured the surface of vertical deformation, referred to as the DS (in m^2^; Fig. 1e). Note that to ensure robustness, we only considered space-scale structures with a vertical deformation greater than 2 m.

### Multiscale analysis of the data

The first step in our approach consisted of the extraction of the significant space-scale structures, as described above, followed by the estimation of the characteristic scales of the different structures.

We primarily processed the acoustic-based lower oxycline depth DS at two grain scales, 40 and 500 m, for the four acoustic surveys (Fig. 2a). We compared ROMS and acoustic lower oxycline depth DS under equivalent conditions (Supplementary Fig. 2) and grain scale (500 m; Fig. 2b).

The same procedure was applied to the acoustically estimated zooplankton biovolume and fish biomass data at a grain scale of 40 m. In these cases, the extracted patterns correspond to space-scale regions of higher abundance (patches). Biological data are characterized by a high skewness, and were thus transformed (cubic root for zooplankton and log(NASC+1) for fish) before the analyses. Day and night data were processed separately (Fig. 2c,d for day and night data, respectively). Seabird GPS track data were collected at daytime only and are thus only presented in Fig. 2c.

We applied a similar wavelet procedure to identify ARS within GPS tracks from seabirds^30^ (Fig. 4c,d). Classical techniques for identifying ARS (First Passage Time^51^) suffer from limitations to handle the multiscale and nested properties of such searching behaviours. The wavelet spectrum is issued from a multiscale analysis of the series of the turning angles observed within the tracks and areas of significant turning rates are isolated. The size of the area encompassed by the ARS is computed as the average diameter of the bounding ellipse.

### Cumulative function of space-scale structures

For each acoustic field considered in this study, we investigated distributions of the structures across-scales. Given the set of extracted significant space-scale structures, we computed the cumulative distributions of each feature (namely, the DS and the zooplankton biovolume and fish biomass) across scales (Fig. 2a–d). For a given feature *W* we proceeded as follows:


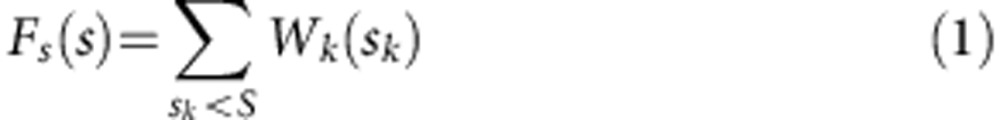


where *S* represents the scale, *k* is the index of the structures, *s*_*k*_ the scale of the *kth* structure and *W*_*k*_ the associated feature (namely, DS and zooplankton biovolume and fish biomass). With a view to comparing the cumulative functions of each feature, we applied the following normalization:


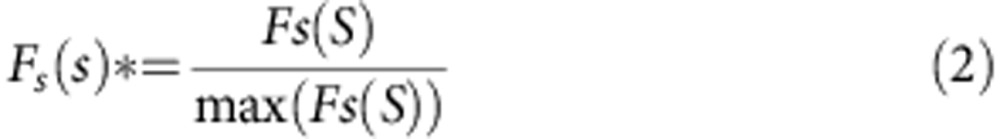


Using a Gaussian kernel smoothing, we computed the density distribution (derivative) of these cumulative distributions with a view to discriminate characteristic scale ranges:


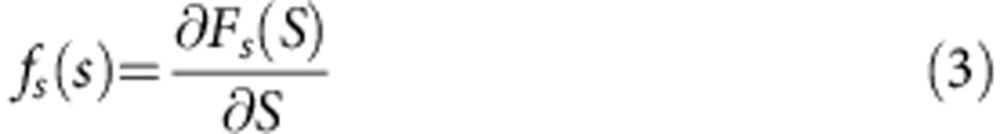


Bootstrap (1,000 replicates) was used to estimate the envelopes of the cumulative and density function of the signal *x* (the DS and the zooplankton biovolume and fish biomass) with a 5% significance level. In the case of ROMS we used 10 output fields and thus we did not bootstrap the data but directly estimated the confidence interval from the 10 fields.

The strong peak at ~300 m in the density function (see Fig. 2c,d) hides the peaks at larger scale. To better depict submesoscale and mesoscale features, we also reported a subplot for scales larger than 1.5 km (see enclosed subplot in Fig. 2c,d).

### Cumulative biomass within DS space-scale structures

To determine the impact of turbulence on zooplankton and fish distributions, we analysed the zooplankton biovolume and fish biomass embedded within physical structures with respect to the characteristic scale of the physical structures (Fig. 3). Turbulence involves hierarchical patterns with small-scale structures embedded within larger ones. These patterns have to be taken into account to avoid replicating the biovolume/biomass in calculations. For that purpose, we define the occupancy signal as:


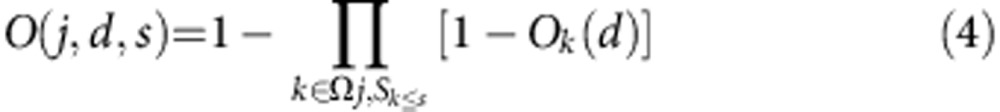


where *j* refers to a transect index, *d* a position along transect *j* and *s* a scale, *Ω*_*j*_ refers to all the physical structures detected along transect *j* and *O*_*k*_(.) is a signal that equals 1 at position along the transect *j* within structure *j* and 0 elsewhere. As such, for transect *j*, *O(j,d,s)* equals 1 if position *d* comprises at least one physical structure with a scale equal or smaller to *s*. Then, the scale-related biovolume/biomass cumulative function is given by:


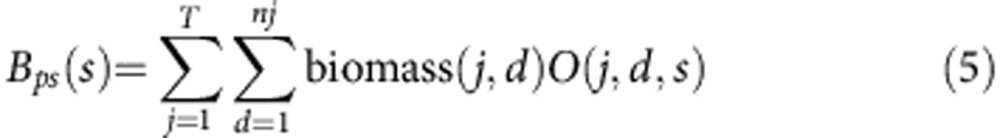


with *T* is the number of survey transects, *n*_*j*_ the number of positions along transect *j* and biomass*(j,d)* is the biomass/biovolume at position *d* above the lower oxycline. As above, we also derived the associated density functions (equation (3)). Furthermore, to study the relative impact of physical processes on biological processes we evaluated the ratio between the density function of zooplankton biovolume (and fish biomass) and DS (see Fig. 3).

### Scale-related aggregation power of physical structures

To estimate the power of aggregation of the physical structures on organisms, we compared the zooplankton and fish density and biovolume/biomass embedded within structures with the zooplankton and fish density and biovolume/biomass observed in the nearest space not forming part of any structure (Table 1). Zooplankton and fish densities were measured as the total biovolume/biomass in the area occupied by the physical structures (respective to a structure-free area) above the lower oxycline. For a given significant physical structure, the associated structure-free area was searched for as the nearest space involving no significant physical structures. Note that such neighbouring structure-free area may not exist in some cases, and thus the corresponding physical structures are not considered in the aggregation index defined hereafter. Let us denote by *S*_*ijk*_ the density for structure *k (k=1,...,M*_*ij*_) along transect *j (j=1,...,Ti)* of survey *i (i=1,...,N)* and *NS*_*ijk*_ the associated density in the associated structure-free area. The mean relative aggregative index (RI) was then computed as the mean of the relative aggregation effects of each physical structure:


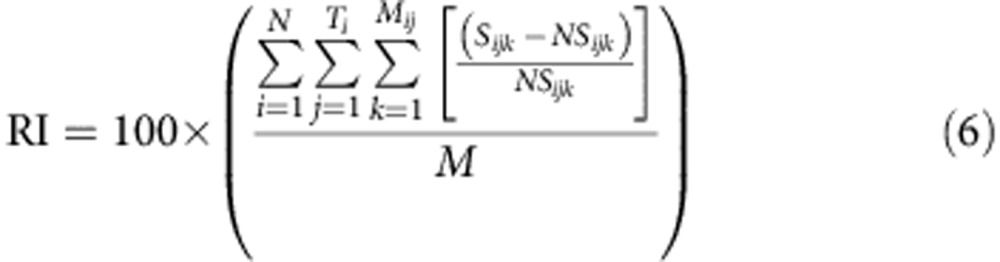


where *N* is the number of survey (*N*=1–4); *T*_*i*_ is the number of transects in survey *i*; *M* is the total number of structures detected from all surveys and transects.

To guarantee robustness to outliers, only values of relative aggregation effect greater (resp. lower) than 5% (95%) percentile of relative aggregation were considered in the evaluation of the mean in equation (6). Unlike zooplankton, fish data encompassed zeros; therefore for fish data, the RI was calculated only when fish density outside of the physical structure was greater than zero.

To test for the significance of the RI (Table 1), we adopted a parametric statistical setting where the null hypothesis assumes statistical independence between the biomass/biovolume signals (that is, biovolume/biomass of zooplankton and fish) and the physical forcing. We estimated the distribution of the RI under the null hypothesis from 500 transect-by-transect random simulations of the ecological signals and the original physical structures. We used a random phase model, with the same second-order structure as the real biomass/biovolume signals^52^. Given the distribution of the RI under the null hypothesis, we evaluated the *P* values of the actual RI values to state the significance of the aggregation power of the physical structures.

## Author contributions

A.B., R.F. and D.G. conceived the study and analyses. A.B., D.G., F.C., S.B., X.C. and R.F. wrote the manuscript. D.G. and G.V. processed the acoustic data. S.B., A.M. and R.F. processed the seabird data. F.C. and X.C performed the modelling work. D.G., S.B., A.C., A.M., A.B. and R.F. carried out statistical analyses.

## Additional information

**How to cite this article:** Bertrand, A. *et al.* Broad impacts of fine-scale dynamics on seascape structure from zooplankton to seabirds. *Nat. Commun.* 5:5239 doi: 10.1038/ncomms6239 (2014).

## Supplementary Material

Supplementary Information Supplementary Figures 1-5, Supplementary Tables 1-2 and Supplementary Note 1

## Figures and Tables

**Table 1:** Scale-related aggregative power of physical processes.

**Scale**	**Zooplankton density**	**Zooplankton biovolume**	**Fish density**	**Fish biomass**
*All structures*				
Mean	8.8**	152.8**	92.0*	947.0**
Median	1.1	76.9	0.5	138.3
*n*	5,878	3,644	4,860	4,860
cv	4.6	1.4	3.4	2.6
				
*<1 km*				
Mean	6.8**	132.5**	61.6**	297.8**
Median	0.6	64.4	0.03	68.0
*n*	3,466	2,957	2,681	2,681
cv	5.4	1.4	3.6	2.1
				
*1–10 km*				
Mean	9.7**	510.4**	111.1**	1,536.8**
Median	2.0	192.8	1.9	243.5
*n*	1,934	1,481	1,721	1,721
cv	4.0	1.7	3.1	2.3
				
*>10 km*				
Mean	26.8**	2,262.1**	318.4**	9,946.7**
Median	2.5	647.9	8.6	799.0
*n*	478	386	4.58	4.58
cv	3.3	1.9	3.1	2.7

cv, coefficient of variation; *n*, number of physical structures.

Difference (in %) between the zooplankton and fish density and biovolume/biomass embedded within structures with the zooplankton and fish density and biovolume/biomass observed in the neighbouring zone with no significant physical structures. To test for significant differences in the relative aggregative index (RI) of the mean density, a parametric statistical setting was used where the null hypothesis assumes statistical independence between the biovolume/biomass of zooplankton and fish and the physical forcing. The distribution of the RI was estimated under the null hypothesis from 500 transect-by-transect random simulations of the ecological signals and the original physical structures. Asterisks indicate significant difference in the mean density: **<0.01, *<0.05;
