# Supplementary material for: Broad impacts of fine-scale dynamics on seascape structure from zooplankton to seabirds
Source: Nat Commun. 2014 Oct 15;5:5239. doi: 10.1038/ncomms6239 (PMC4218954; doi:10.1038/ncomms6239)
Supplement: Supplementary Information — Supplementary Figures 1-5, Supplementary Tables 1-2 and Supplementary Note 1 [file ncomms6239-s1.pdf]

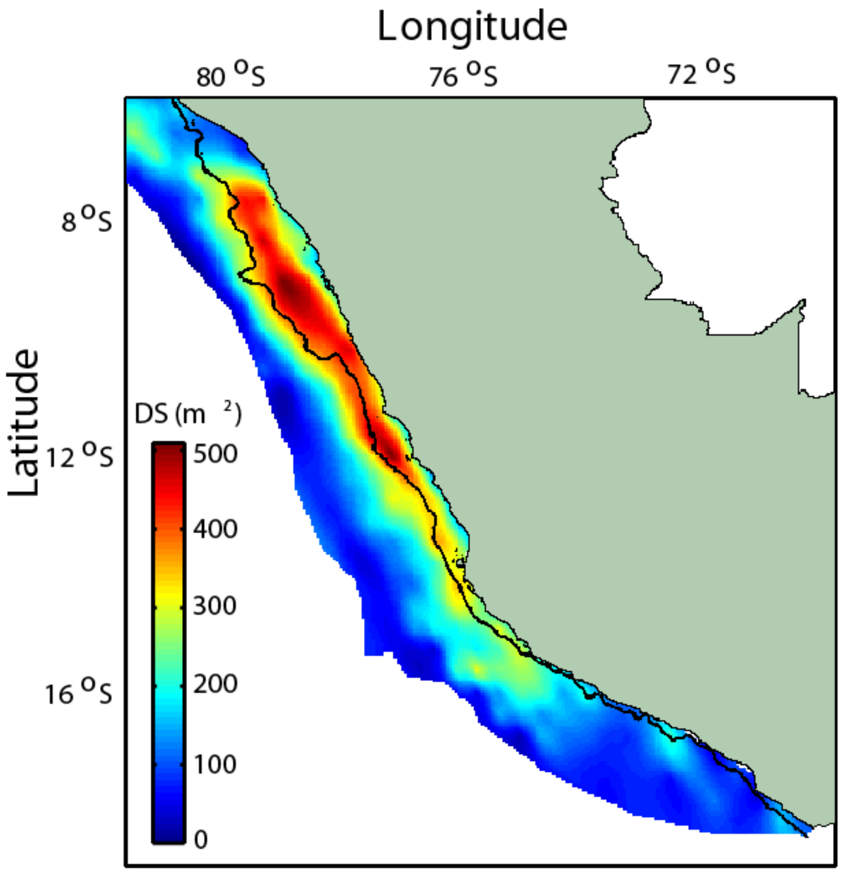

Supplementary Figure 1 | Space-scale structures structure distribution. Interpolated map of distribution of the downward deformation surface (DS) of the space-scale structures with a scale less than 1 km (the black line drawn represents the shelf break).

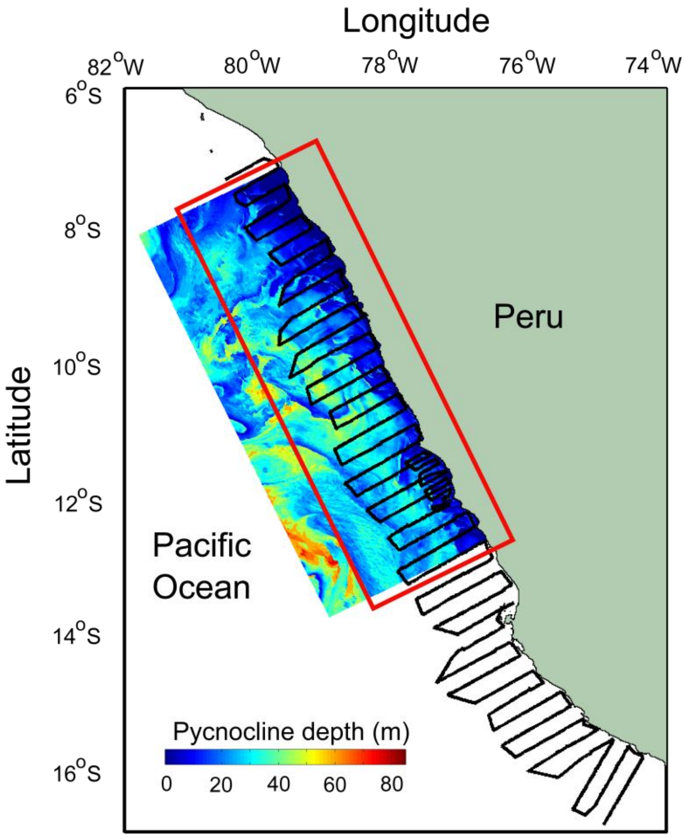

Supplementary Figure 2 | ROMS configuration. Pycnocline depth (colour shading) from the ROMS run performed for austral spring conditions and the path of the acoustic survey performed in November-December 2008 (black solid line). The red box shows the area used to compare acoustic and ROMS.

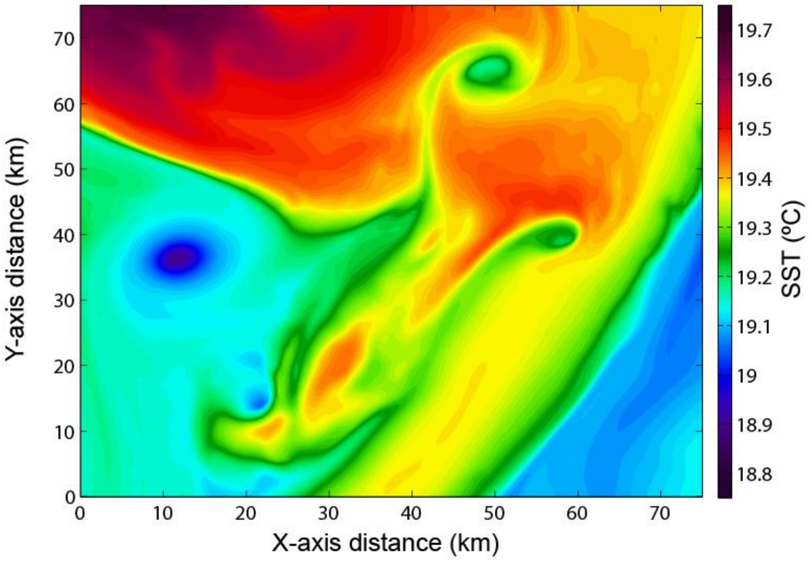

Supplementary Figure 3 | ROMS output. Snapshot of modelled sea surface temperature (SST in ºC) exhibiting submesoscale coherent eddies and filaments off the coast of central Peru. The model grid horizontal resolution is 500 m.

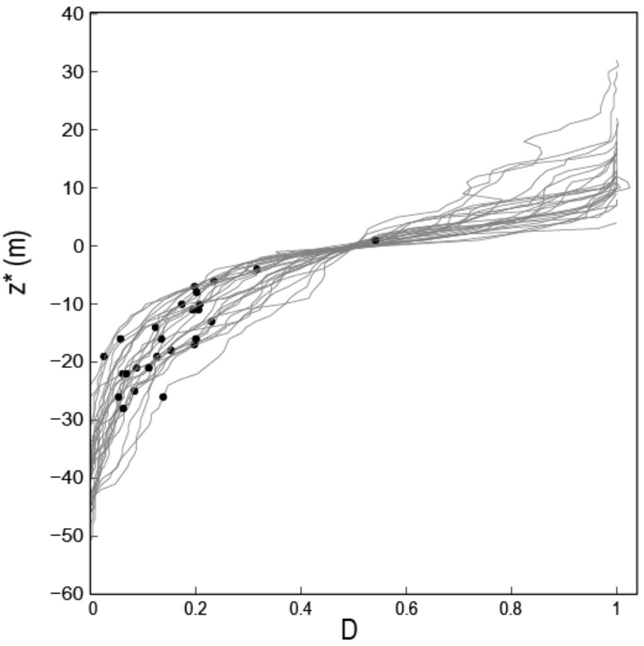

Supplementary Figure 4 | Lower oxycline depth relationship with an isopycnal surface. Water density profiles (grey solid line) for $D=(\rho-\rho_{(60\ m)})/(\rho_{(0\ m)}-\rho_{(60\ m)})$ as a function of $z^*=z-z(D=0.5)$; D=0.5 matching the region of largest vertical density gradient, i.e. the pycnocline location. In each profile, the black dots indicate the positions of the lower oxycline as inferred from acoustics.

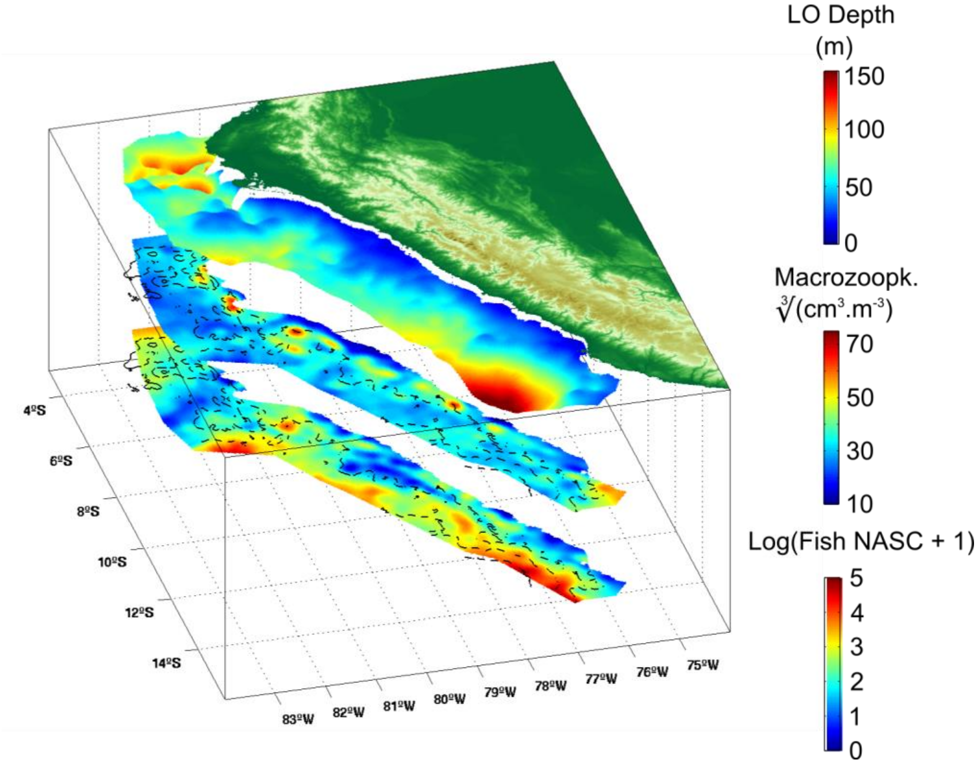

Supplementary Figure 5 | Data description, night conditions in Austral Spring 2010. The upper surface shows the acoustically estimated depth of the lower oxycline (LO, in m). The intermediate surface shows the acoustically estimated macrozooplankton biovolume (in mm^3 m^{-2}) present above the oxycline. The lower surface shows the acoustically estimated pelagic fish biomass (nautical area scattering coefficient, NASC, in m^2 nm^{-2}) present above the oxycline. Note that during the night the zooplankton and fish present in the oxygenated surface volume belong to the epipelagic plus the migrant mesopelagic communities.

Supplementary Table 1 | Characteristic scales (in km) of turbulent structures.

Scale	Acoustic (40 m) All data	Acoustic (40 m) ROMS Area	Acoustic (500 m) ROMS Area	ROMS (500 m) ROMS Area
Scale 1	0.17-0.67	0.17-0.51	n/a	n/a
Scale 2	1.4-3.7	1.3-2.6	1.6-3.7	1.9-5.5
Scale 3	7.3-12.9	4.1-9.7	6.1-10.7	9.7-15.6
Scale 4	22.8-33.4	22.8-33.4	22.8-36.8	25.1-36.8

Characteristic scales were identified from local maxima i.e. the zero-crossings of the second derivative of the density function of the lower oxycline deformation surface (DS) estimated from acoustic data (grain scale: 40 m) from the complete set of data, and from acoustic lower oxycline (grain scales: 40 and 500 m) and ROMS pycnocline depth (grain scale= 500 m) in the ocean area modelled by ROMS (Figure S2).

Supplementary Table 2 | Characteristic scales (in km) of turbulent structure zooplankton and fish patch and seabird ARS.

Scale	DS Day+Night	DS Day	Zooplankton Day	Fish Day	Booby Day	Guanay Day	DS Night	Zooplankton Night	Fish Night
Scale 1	0.17-0.67	0.17-0.67	0.12-0.34	0.12-0.31	0.19–0.46	0.26–0.76	0.17-0.81	0.12-0.31	0.12-0.31
Scale 2	1.4-3.7	1.4-4.5	1.3-3.1	1.3-2.8	1.4-3.5	1.3-3.2	1.4-3.4	1.3-2.8	1.3-3.0
Scale 3	7.3-12.9	8.0-12.9	12.9-17.2	8.8-14.2	4.4-8.5	5.3-11.0	6.6-12.9	4.1-6.0	7.3-9.7
Scale 4	22.8-33.4	22.8-36.8	30.4- 33.4	27.6-40.5	18.5-25.9	17.6-23.5	20.7-33.4	15.6-33.4	22.8-36.8

Characteristic scales were identified from local maxima i.e. the zero-crossings of the second derivative of the density function of the lower oxycline deformation surface (DS), the zooplankton and fish biovolume/biomass estimated from acoustic data, and the seabird ARS diameter in the complete data set (grain scale=40 m).

Supplementary Note 1 | Supplementary information for Figure 4 On November 25, 2011 the R/V was cruising in the area where seabird tagging was ongoing (Pescadores Island, 11°46'S - 77°15'W; ~7 km off the coast). To increase the acoustic spatial coverage in this area, the inter-transect distance was set to 5 nm instead of 15 nm. A tracked Guanay cormorant flew across the survey path 30 minutes after the R/V passed through and foraged within a submesoscale structure detected using acoustic data. This adult Guanay weighting 2025 g, performed a foraging trip from 11:09 am to 01:52 pm local time. The total distance travelled was 59.9 km at an average flight speed of 13.7 m s^{-1}.
